# RNA-seq of life stages of the oomycete *Phytophthora infestans* reveals dynamic changes in metabolic, signal transduction, and pathogenesis genes and a major role for calcium signaling in development

**DOI:** 10.1186/s12864-017-3585-x

**Published:** 2017-02-23

**Authors:** Audrey M. V. Ah-Fong, Kyoung Su Kim, Howard S. Judelson

**Affiliations:** 1 Department of Plant Pathology and Microbiology, University of California, Riverside, CA 92521 USA; 2 Present address: Department of Applied Biology, College of Agriculture and Life Sciences, Kangwon National University, Chuncheon, Korea

**Keywords:** Oomycete, Spore development, Zoospore, Gene regulation, Transcriptomics

## Abstract

**Background:**

The oomycete *Phytophthora infestans* causes the devastating late blight diseases of potato and tomato. *P. infestans* uses spores for dissemination and infection*,* like many other filamentous eukaryotic plant pathogens. The expression of a subset of its genes during spore formation and germination were studied previously, but comprehensive genome-wide data have not been available.

**Results:**

RNA-seq was used to profile hyphae, sporangia, sporangia undergoing zoosporogenesis, motile zoospores, and germinated cysts of *P. infestans.* Parallel studies of two isolates generated robust expression calls for 16,000 of 17,797 predicted genes, with about 250 transcribed in one isolate but not the other. The largest changes occurred in the transition from hyphae to sporangia, when >4200 genes were up-regulated. More than 1350 of these were induced >100-fold, accounting for 26% of total mRNA. Genes encoding calcium-binding proteins, cation channels, signaling proteins, and flagellar proteins were over-represented in genes up-regulated in sporangia. Proteins associated with pathogenicity were transcribed in waves with subclasses induced during zoosporogenesis, in zoospores, or in germinated cysts. Genes involved in most metabolic pathways were down-regulated upon sporulation and reactivated during cyst germination, although there were exceptions such as DNA replication, where transcripts peaked in zoospores. Inhibitor studies indicated that the transcription of two-thirds of genes induced during zoosporogenesis relied on calcium signaling. A sporulation-induced protein kinase was shown to bind a constitutive Gβ-like protein, which contributed to fitness based on knock-down analysis.

**Conclusions:**

Spore formation and germination involves the staged expression of a large subset of the transcriptome, commensurate with the importance of spores in the life cycle. A comparison of the RNA-seq results with the older microarray data indicated that information is now available for about twice the number of genes than before. Analyses based on function revealed dynamic changes in genes involved in pathogenicity, metabolism, and signaling, with diversity in expression observed within members of multigene families and between isolates. The effects of calcium signaling, a spore-induced protein kinase, and an interacting Gβ-like protein were also demonstrated experimentally. The results reveal aspects of oomycete biology that underly their success as pathogens and potential targets for crop protection chemicals.

**Electronic supplementary material:**

The online version of this article (doi:10.1186/s12864-017-3585-x) contains supplementary material, which is available to authorized users.

## Background

Microbes rely on the well-orchestrated regulation of cellular and developmental processes to complete their life cycles, which in filamentous plant pathogens typically involve transitions between mycelia, spores, and infection structures [1, 2]. Over past decades, transcriptional changes during the life cycle have been identified by a parade of technologies. These include subtraction cDNA cloning, mRNA differential display, macroarrays, microarrays, and more recently RNA-seq. Each new technology has offered the prospect of more accurate and comprehensive coverage of the transcriptome, reaching maximum impact when paired with an annotated genome.

In 2008, we reported on the mRNA content of asexual life stages of the oomycete *Phytophthora infestans,* the cause of the devastating late blight diseases of potato and tomato [3]. Using potato isolate 88069, microarrays based on 15,650 unigenes mostly derived from cDNAs revealed that the transcript abundance of about half of unigenes changed significantly during the life cycle. This involved comparing nonsporulating hyphae, asexual sporangia (which are hydrated and metabolically active), sporangia stimulated to cleave into zoospores by chilling, zoospores released from the sporangia, and germinated zoospore cysts. Similar stages are made by most oomycetes, and are critical to their dissemination and pathogenic success [4, 5]. Zoospores are especially important since these biflagellated chemotactic cells help the pathogen reach the plant and locate optimal infection sites [6]. Zoosporogenesis is also intriguing due to its rapidity; sporangial cytoplasm starts cleaving into individual zoospores within minutes after exposure to water, generally at cool temperatures which favor zoospore survival. *De novo* transcription and translation are not needed for zoospore release, since all required proteins pre-exist in sporangia [7]. Secondary messengers such as calcium help regulate zoosporogenesis and later stages [8].

Differentially expressed genes identified by the 2008 study included many potential regulators such as protein kinases and phosphatases, metabolic enzymes that may mobilize reserves and maintain energy homeostasis, structural proteins, and pathogenicity factors [3]. However, limitations of the microarray study became apparent after the *P. infestans* genome sequence was released in 2009 [9]. For example, only about two-thirds of the 17,797 genes annotated in the genome were represented on the microarray, and the same gene was often represented by multiple unigenes. Functional annotations were incomplete or misleading, since unigenes often contained only part of the protein-coding sequence. Many unigenes were also derived from transposable elements. In addition, the accuracy of expression calls was limited by the dynamic range of the Affymetrix microarray technology used in the 2008 study. None of these issues are unique to *P. infestans,* as they simply reflect the maturation of technologies and genome resources available for any system [10].

The goal of the present study was to update our understanding of the *P. infestans* transcriptome using RNA-seq with its annotated whole-genome sequence. Using an isolate from tomato, 1306, we analyzed mRNA from vegetative hyphae, sporangia, cleaving (chilled) sporangia, zoospores, and germinated cysts. We also sequenced mRNA from isolate 88069 that had been used in the 2008 study, which confirmed the results from isolate 1306 and allowed the RNA-seq and microarray results to be compared. Robust expression calls were made for approximately 16,000 genes in RNA-seq compared to 7584 in the microarrays, and the number of differentially expressed genes increased proportionally. We also extended the transcriptomic analysis beyond the 2008 study by identifying genes expressed during zoosporogenesis that are controlled by calcium signaling, studying a protein interacting with a protein kinase induced during sporulation, and identifying expression polymorphisms between 1306 and 88069.

## Results and Discussion

### RNA sequencing strategy

Using Illumina technology, 75-nt single-end sequence data were obtained from isolates 1306 and 88069 using a minimum of two biological replicates*.* The 1306 material was prepared specifically for this study, and included nonsporulating mycelia from rye-sucrose media, purified sporangia, sporangia chilled for 60 min to initiate zoosporogenesis, zoospores, and zoospore cysts germinated for 6 h in water. About half of the germ tubes in the latter contained swellings resembling appressoria formed *in planta*. We also sequenced the same RNA from isolate 88069 that had been used in the microarray study, except for RNA from germinated cysts. A total 674 million reads were obtained, averaging 24.1 million per sample with a minimum of 20 million passing quality control for each biological replicate (Q > 30; Additional file 1). The fraction of reads mapping to *P. infestans* gene models averaged 87.3%. As discussed later, robust expression calls were obtained for about 16,000 genes per isolate.

### Overview of changes between life-stages

**Fig. 1:**
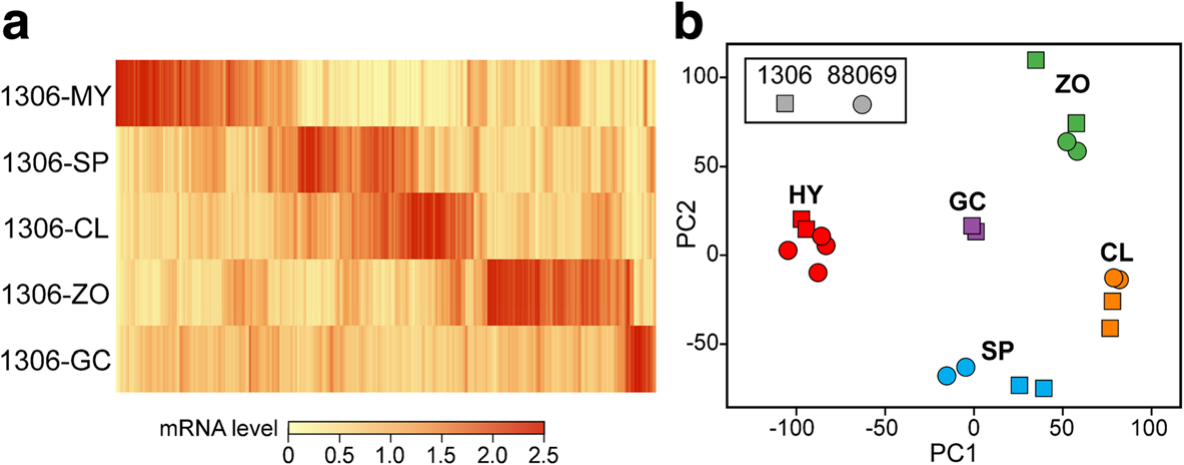
Overview of mRNA during development. **a** heatmap of mRNA levels in isolate 1306 from non-sporulating mycelia (MY), purified sporangia (SP), sporangia chilled in water to induce the cleavage of sporangia into zoospores (CL), zoospores released from the sporangia (ZO), and germinated cysts (GC). Cartoons illustrating each stage are shown in Fig. 2. Data are TMM-normalized CPM values subjected to per-gene normalization and then hierarchical clustering using the Euclidean average linkage method. **b** Principal component analysis of samples from isolates 1306 (*squares*) and 88069 (*circles*), labelled as in panel **a**. The data in both panels were filtered to only include genes with CPM ≥ 1.0

**Fig. 2:**
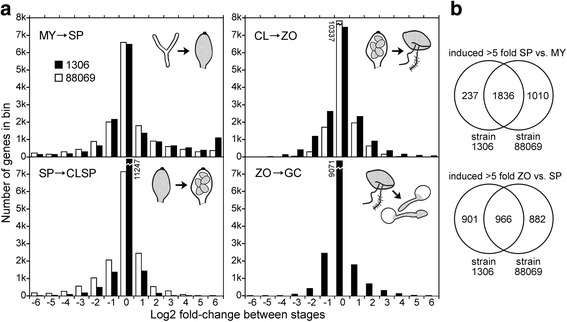
Number of genes changing at developmental transitions. **a** Data from isolate 1306 (*black bars*) and isolate 88069 (*white bars*) showing changes between mycelia (MY), sporangia (SP), chilled sporangia (cleaving, CL), and germinated cysts (GC). In the upper-left graph, for example, the bar in the bin labelled 5 represents the number of genes showing more than 2^5^ but less than 2^6^-fold higher mRNA levels in sporangia than mycelia. **b** Comparison of genes induced ≥5-fold in sporangia versus mycelia, and zoospores versus sporangia, of the two isolates

Large changes in the transcriptome were observed at each developmental transition. A heatmap based on TMM-normalized CPM (counts per million) data from 1306 is shown in Fig. 1a, a principal component analysis (PCA) plot comparing 1306 and 88069 is in Fig. 1b, and cartoons illustrating each stage are shown in Fig. 2a. CPM values, fold-change ratios, and false discovery rate (FDR) statistics based on the Bonferroni-Hochberg method are in Additional files 2 and 3.

The number of genes changing at each life-stage transition in 1306 and 88069 are graphed in Fig. 2a. Based on a FDR cut-off of 0.05, an average of 4876 and 4286 genes (30 and 26% of all expressed genes) were up and down-regulated in the transition between mycelia and sporangia in the two isolates, respectively. The largest differences were between mycelia and sporangia. Up-regulated in sporangia versus mycelia by >5-fold were 2073 and 2846 genes in isolates 1306 and 88069, respectively. Up-regulated by >5-fold in both isolates were 1836 genes (Fig. 2b). Differences between isolates were due mostly to the fold-change threshold, *i.e.* a gene induced 5.5-fold in 1306 and 4.5-fold in 88069 would not fall in the overlapping gene set. PCA analysis indicated strong similarity between the isolates (Fig. 1b).

When comparing zoospores to sporangia, 1867 and 1848 genes were up-regulated by >5-fold in isolates 1306 and 88069, respectively, with 966 genes (52%) common to both (Fig. 2b). Some of the discrepancy between strains in this comparison is likely due to differences in the timing and efficiency of zoospore release, which varies between experiment and isolate. Also, while 1306 produces nearly exclusively mononucleate zoospores, in our hands about 20% of 88069 sporangia do not fully cleave and form multinucleate zoospores.

Expression level polymorphisms caused by changes in promoters, deletions, or epigenetic silencing could also explain some interisolate differences. At the extreme, this was manifested in cases where genes were expressed in only one isolate. For example, 195 genes that appeared unexpressed in isolate 1306 (CPM < 1.0) exhibited CPM values higher than 10 in isolate 88069; the threshold of 10 was selected to avoid false positives, since that value is close to the median of all genes. By the same criteria, 95 genes scored as being expressed in isolate 1306 but not 88069. Of the 195 genes for which expression was detected only in 88069, a search against a genome assembly of 1306 indicated that in 162 cases complementary sequences were present. Therefore, gene deletion does not appear to be the main cause of a lack of expression in 1306. Nine of the 195 genes were annotated as RXLR effectors, which are known to be subject to epigenetic silencing [11].

There were also cases where a gene up-regulated in one isolate during a developmental transition was down-regulated in the other. For example, of 2073 genes that had >5-fold higher mRNA in sporangia of isolate 1306 compared to mycelia, 27 had less mRNA in sporangia of 88069 versus mycelia. Interestingly, 24 of these 27 genes belonged to multigene families. Different family members may have evolved distinct patterns of expression, as observed within families in other systems [12].

Overall, the data indicate that *P. infestans* devotes much resources to spores, when considering the amount of new RNA (and presumably protein) made during sporulation. The number of genes having mRNA levels >100-fold and >10-fold higher in at least one spore stage (sporangia, chilled sporangia, or zoospores) compared to hyphae was 1355 and 3520, respectively. This corresponded to 8.5 and 22% of all expressed genes. The number of reads from “spore-specific” genes, *i.e.* those induced >100-fold in spores compared to hyphae, comprised 26% of all reads. The average CPM of spore-specific transcripts was three-fold higher than the average gene.

### RNA-seq versus microarray results

To compare the RNA-seq and Affymetrix data, the 15,650 unigenes used to design the Affymetrix features were compared to the 17,797 genes in the *P. infestans* genome assembly. The unigenes were assigned to a gene if their sequence similarity was ≥96%; this threshold was used to account for the reference genome being from a different strain of *P. infestans* than 1306 or 88069. It was not possible to match 646 unigenes to genes due to ambiguity resulting from multigene families; we deemed this to be the case where the percent match between two genes and the unigene were within 4% of each other. In addition, 2051 unigenes did not match any gene. Most of the latter were derived from transposable elements or noncoding RNAs. In total, 10,468 *P. infestans* genes were matched to the unigenes.

We next compared the number of “expressed” genes. For RNA-seq, genes with FPKM (fragments per kilobase of transcript per million mapped reads) values ≥1.0 were defined as expressed. This is a conservative threshold that corresponds to about 22 reads per average-sized gene per sample. By this criteria, 15,588 and 15,839 genes were expressed in 1306 and 88069, respectively. An alternative threshold of 10 mapped reads per gene (regardless of size) defines 15,974 and 16,150 genes as expressed in the two isolates, respectively. For Affymetrix data, we used the assignment of a present flag by MAS 5.1 software to signify expression. This indicated that 7584 genes were detected on the microarrays. Thus, RNA-seq detected at least 210% more expressed genes than the Affymetrix platform.

**Fig. 3:**
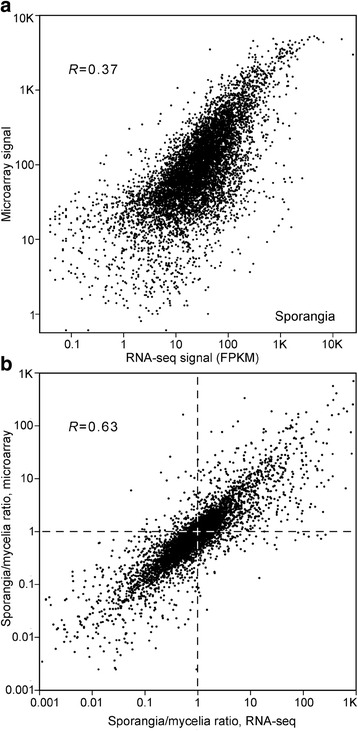
Comparisons of Illumina RNA-seq and Affymetrix microarray data. **a** Expression level calls of mRNA from sporangia. Data are based on the analysis of identical preparations of mRNA from isolate 88069. Pearson’s correlation coefficient (R) was calculated from genes with FPKM > 1. **b** Fold-change values of genes in sporangia versus mycelia, based on expression calls from the two technologies

To illustrate the relationship between expression level from RNA-seq and the microarrays, data from 88069 sporangia are shown in Fig. 3a. This is based on the analysis of identical RNA. There is a positive correlation, but with compression of the Affymetrix signal evident for genes with FPKM > 2000 and major scatter at FPKM < 100. We also compared the ratio of expression in sporangia versus hyphae, again based on the same RNA samples from isolate 88069. There was moderate correlation between the RNA-seq and Affymetrix results (*R* = 0.63; Fig. 3b). The concordance between the two technologies is slightly less than that reported for similar comparisons in other taxa [13, 14].

### Gene Ontology (GO) analysis

To obtain insight into the types of genes that change during the life cycle, GO term enrichment analysis was performed (Table 1). Results for 1306 and 88069 were similar. The most over-represented term in genes up-regulated in sporangia compared to hyphae was cilium (GO:0005929), with FDR values of 10^-41^ and 10^-38^ in 1306 and 88069, respectively. This term reflects the induction of genes for the flagellar axoneme and basal body. Another over-represented term was signaling (GO:0023052), due to many sporangia-induced protein kinases, transcription factors, and ion channels. The term for cytoskeleton (GO:0005856) was also over-represented due to many kinesins, dyneins, and proteins with interaction modules such as ankyrin or WD40 domains. Other notable terms were cation channel, calcium binding, and cyclic nucleotide metabolism. Sporangia-induced genes in the latter category include those encoding cAMP and cGMP-regulated protein kinases and phosphodiesterases.

**Table 1:** Over-represented GO terms common to 1306 and 88069 development^a^

Stage	GO Term	Definition	Type^b^	1306 FDR^c^	88069 FDR^c^
Sporangia vs. hyphae	GO:0005929	cilium	CC	1.8E-41	3.3E-38
	GO:0023052	signaling	BP	2.2E-25	1.0E-16
	GO:0005856	cytoskeleton	CC	2.2E-24	5.7E-30
	GO:0000902	cell morphogenesis	BP	7.9E-19	3.0E-21
	GO:0005886	plasma membrane	CC	1.3E-18	6.3E-11
	GO:0004674	protein serine/threonine kinase	MF	2.1E-14	1.5E-09
	GO:0005261	cation channel activity	MF	6.1E-13	5.8E-11
	GO:0003774	motor activity	MF	1.1E-09	9.5E-09
	GO:0005524	ATP binding	MF	1.9E-08	1.0E-07
	GO:0009187	cyclic nucleotide metabolic process	BP	1.7E-07	2.7E-04
	GO:0005509	calcium ion binding	MF	4.2E-06	2.7E-04
	GO:0004872	receptor activity	MF	1.3E-04	7.3E-05
	GO:0008092	cytoskeletal protein binding	MF	6.6E-03	1.4E-04
Zoospore vs. sporangia	GO:0044426	cell wall part	CC	1.7E-10	9.3E-07
	GO:0004674	protein serine/threonine kinase	MF	6.6E-10	7.2E-09
	GO:0016837	carbon-oxygen lyase, polysaccharide	MF	2.8E-06	2.8E-06
	GO:0030570	pectate lyase activity	MF	1.8E-05	1.8E-05
	GO:0050525	cutinase activity	MF	1.8E-05	4.9E-04
	GO:0004748	ribonucleoside-diphosphate reductase	MF	1.3E-03	1.3E-03
	GO:0016835	carbon-oxygen lyase activity	MF	4.7E-03	4.7E-03
Germinating cysts vs. sporangia	GO:0000502	proteasome complex	CC	5.9E-11	—
	GO:0004553	glycosyl hydrolase activity	MF	3.8E-08	—
	GO:1901575	organic substance catabolic process	BP	3.1E-06	—
	GO:0016054	organic acid catabolic process	BP	6.0E-06	—
	GO:1902221	PEP family amino acid metabolism	BP	2.9E-05	—
	GO:0005975	carbohydrate metabolic process	BP	3.5E-05	—
	GO:1901565	organonitrogen compound catabolism	BP	3.7E-05	—
	GO:0005576	extracellular region	CC	1.1E-04	—
	GO:0031988	membrane-bounded vesicle	CC	6.3E-04	—
	GO:0005759	mitochondrial matrix	CC	1.1E-03	—
	GO:0003868	4-hydroxyphenylpyruvate dioxygenase	MF	2.6E-03	—
	GO:0045454	cell redox homeostasis	BP	3.1E-03	—
	GO:0016885	ligase, forming carbon-carbon bonds	MF	4.9E-03	—
	GO:0016052	carbohydrate catabolic process	BP	5.9E-03	—
	GO:0004650	polygalacturonase activity	MF	6.0E-03	—
	GO:0004298	threonine-type endopeptidase	MF	7.6E-03	—

^a^ After removing redundant terms and merging groups having >75% of genes in common

^b^ BP, biological process; MF, molecular function; CC, cellular component

^c^ False discovery rate, corrected for multiple testing by Benjamini-Hochberg method

GO terms associated with genes induced in zoospores compared to sporangia include protein kinase (which was also in the sporangia-induced list), pectate lyase, and cutinase. The latter two are needed to degrade the plant cell wall during infection, so it appears that the developmental program anticipates their need in the subsequent germinated cyst/appressoria stage. A related argument can be made to explain the over-representation of ribonucleoside diphosphate reductase in the zoospore-induced genes. This enzyme forms deoxyribonucleotides, which are needed to support the resumption of nuclear division after cyst germination [15].

Two other GO terms related to plant cell wall degradation, polygalacturonase and glycosyl hydrolase, were associated with genes induced in germinated cysts compared to sporangia. However, most terms associated with germinated cysts are linked to general metabolism. These include mitochondrial matrix enzymes, proteins involved in redox homeostasis, and proteosomal proteins. As will be noted later, this reflects the fact that many such genes are down-regulated during sporulation and then turned on again in germinated cysts.

**Fig. 4:**
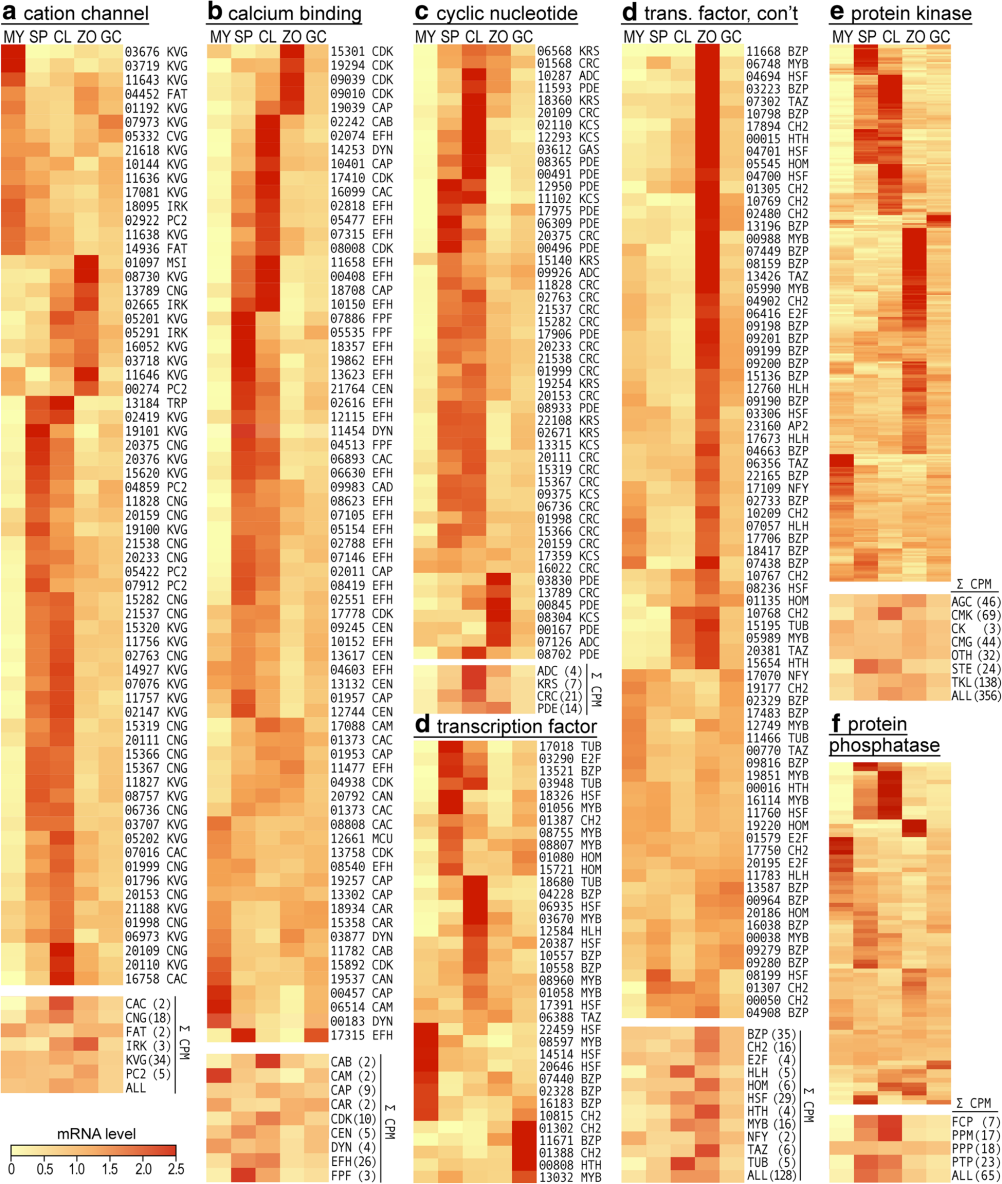
Expression patterns of major classes of regulatory genes. Indicated are data from nonsporulating mycelia (MY), sporangia (SP), cleaving sporangia (CL), zoospores (ZO), and germinated cysts (GC) of isolate 1306. In most panels, the genes are labeled with their five-digit gene number (*i.e.* 03676) and an abbreviation for the functional subclass. Labels are not shown for protein kinases and phosphatases due to space limitations, but the data are in Additional file 3. Below most panels are small heatmaps that indicate the sum of CPM values for genes in subclasses (Σ CPM, with the number of genes in parentheses), or all genes (ALL). Gene numbers not shown are presented in Additional file 5. **a** cation channel. **b** calcium binding. **c** cyclic nucleotide. **d** transcription factor. **e** protein kinase. **f** protein phosphatase

Figure 4 portrays the expression of several categories of regulatory genes in isolate 1306, several of which were highlighted by the GO term analysis. Each gene is marked with its five-digit number, which is abbreviated from the PITG_NNNNN nomenclature used in the Broad Institute’s annotation of *P. infestans.* Most are also appended with codes denoting function, which are defined in the figure legend. In this and other figures, only genes with CPM ≥ 1.0 are shown.

### Cation channels

This GO term was over-represented in sporangia-induced genes in both isolates 1306 and 88069, with FDR < 10^-11^. When all genes encoding cation channels were examined, about three-quarters were found to be transcribed preferentially in sporangia, cleaving sporangia, or zoospores (Fig. 4a). About one-quarter are expressed mostly in hyphae, and none in germinated cysts. In the figure, they are classified into six major families based on schemes used in other eukaryotes [16]. Most of the six families include both hyphal and spore-dominant forms with the exception of the FAT group, which is expressed primarily in hyphae and encode H^+^-transporting F-ATPases. The spore-induced genes encode members of the calcium, cyclic nucleotide-gated, potassium voltage-gated, and polycystin 2 (PC2) channel families. Since polycystin 2 is a thermosensitive calcium channel in animals, the two sporangia-induced PC2 genes of *P. infestans* might regulate zoosporogenesis [17].

While traditional heatmap analysis is useful for detecting trends in gene expression, it does not reflect the amount of transcripts from each gene or subclass. Therefore, shown at the bottom of the cation channel column in Fig. 4a are the sum CPM values (Σ CPM) of genes in each category, and all cation channels. This shows that the total CPM of all transcripts for cation channels nearly double in spores compared to mycelia, largely due to the 28 and 5-fold induction of calcium (CAC) and inward rectifier potassium channels (IRK), respectively. The aggregated CPM of all cation channels increases slightly in spores; their peak level is in chilled sporangia is 79% higher than in hyphae.

### Calcium-binding proteins

Possibly linked to the increased mRNA abundance of calcium channel genes in spores is the concomitant induction of more than half of all genes encoding calcium-binding proteins (Fig. 4b). The corresponding GO term was over-represented in sporangia-induced genes in both isolates 1306 and 88069, with FDR values of 10^-6^ and 10^-4^, respectively. Most genes encoding calcium-binding proteins are expressed chiefly in sporangia and cleaving sporangia, with a few induced in zoospores or hyphae. No gene in this group exhibits peak expression in germinated cysts. Eukaryotes produce more than 45 families of calcium-binding proteins [18], and we could classify the *P. infestans* proteins into more than ten of these.

One class that is transcribed preferentially in spores encodes centrin (CEN). Three of the four predicted centrin genes are up-regulated strongly in sporangia, consistent with the protein’s role in the flagella-anchoring basal body [19]. The other predicted centrin, PITG_13121, is expressed the most in hyphae, which may signal that it is used to assemble the centrosome for mitosis.

Since protein kinase inhibitors block zoosporogenesis [20], it was interesting to observe that multiple calcium-mediated pathways for regulating kinases were induced in spores. One involves kinases that bind calcium directly, which are named CDK in Fig. 4b. Their aggregate CPM values peak in cleaving sporangia, reflecting the pattern of seven of the eight CDKs. PITG_15892 is however transcribed chiefly in hyphae. A second pathway that regulates kinases involves the Mo25/CAB39 family (CAB). While CDKs bind calcium directly, Mo25/CAP39 binds calcium and then regulates STE20 kinases [21]. *P. infestans* contains two genes that encode Mo25 proteins; one is constitutive while the other, PITG_02242, is induced 10-fold in cleaving sporangia compared to hyphae. A third calcium-mediated mechanism for regulating protein kinases is represented by the calmodulin-dependent protein kinase family, of which most were induced in sporangia. These are not shown in Fig. 4b as they will be discussed later along with other protein kinases. While the canonical calmodulin gene (CAM) has 5-fold higher levels in hyphae than sporangia, several calmodulin-like proteins are spore-induced.

A diversity of expression patterns were observed in the nine genes encoding calcium-dependent protein phosphatases, also known as calcineurin (CAP). Different members of this group showed higher mRNA levels in sporangia, zoospores, cleaving sporangia, and hyphae. While three calcineurin genes were expressed the most in hyphae, total CPM values of the family peaked in cleaving sporangia at two-fold the level of hyphae.

Twenty-five predicted calcium-binding proteins were difficult to categorize functionally, as their main defining features were EF hands. These are thus marked as EFH proteins in Fig. 4c. Nearly all were induced strongly in sporangia or cleaving sporangia, and encoded two EF hands. PITG_02551 and PITG_02788 were deemed calmodulin-like since they were similar in size to the canonical protein (PITG_06514), although they diverged in amino acid sequence by 35 and 41%, respectively. Two proteins with exceptional structures were PITG_10150 and PITG_04603, which encoded a remarkable 8 and 10 EF-hands, respectively. While PITG_04603 expression was fairly constitutive, PITG_10150 was induced >100-fold in sporangia compared to hyphae, with an apparently short mRNA half-life since virtually no signal was detected in zoospores. Both zoosporic and azoosporic oomycetes encode orthologs of these novel proteins.

Several categories of calcium-binding proteins show reduced mRNA in spores. One example is the single canonical calmodulin gene (CAM), which is >5-fold higher in hyphae. Also expressed most in hyphae are all three genes encoding calreticulin (CAR), which helps proteins fold in the endoplasmic reticulum after translation [22]. Its decline in spores mirrors the decline in mRNAs for genes involved in translation and many other general metabolic pathways, which will be discussed later. After falling in sporangia, calreticulin transcripts rise in germinating cysts which is consistent with the resumption of growth. The same descent and recovery of mRNA levels was observed when measuring the total CPM of dynamin (DYN), a GTPase involved in vesicle formation and cytokinesis [23]. Two genes encoding dynamin were nevertheless induced in sporangia, where they might play roles in zoospore cleavage.

Gene PITG_12661, which encodes a mitochondrial calcium uptake protein, was expressed at slightly higher levels in hyphae than spores. A similar pattern was observed for genes encoding most non-metabolic mitochondrial proteins that did not bind calcium, such as the ftsZ cell division protein (PITG_03373), the mitochondrial protein import receptor TOM40 (PITG_13250), and ribosomal proteins (*e.g.* S15, PITG_12839). The interpretation is that mitochondrial biogenesis may be down-regulated in spores. This seems to contradict the results of a prior study, which reported that the ratio of mitochondrial to cytoplasmic rDNA rises in zoospores [24].

### Cyclic nucleotide-related proteins

Based on gene expression pattern, cAMP and/or cGMP seem to be additional secondary messengers that are active in spores (Fig. 4c). Genes in the category include the catalytic and regulatory domains of adenylyl (adenylate) or guanylyl cyclases, phosphodiesterases, cyclic nucleotide-regulated protein kinases, and cyclic nucleotide-gated ion channels. Nearly all are expressed mostly in sporangia, cleaving sporangia, and zoospores, with very low levels in hyphae (Fig. 4c). Only two genes in the category are expressed constitutively; these encode a predicted protein kinase subunit (PITG_17359) and a cAMP receptor (PITG_16022).

Based on CPM totals, the genes encoding the catalytic and regulatory subunits of nucleotidyl kinases have nearly identical expression profiles, with their mRNAs peaking in cleaving sporangia at >100 and 34-fold higher levels than in hyphae. While all three catalytic enzyme genes are induced in spores, 99% of the counts come from PITG_10287. Based on residues in the nucleotide-binding pocket [25], PITG_09926 seems to be a guanylyl kinase, but the specificity of PITG_07126 and PITG_10287 can not be predicted.

While the data suggest that cyclic nucleotides play a role in the life cycle, a prior study indicated that cAMP and cGMP levels do not change during zoosporogenesis [26]. Whether the compounds play a role in sporulation, zoospore swimming, or cyst germination is not known.

### Transcription factors

Shown in Fig. 4d are sequence-specific transcription factors. About three-quarters are transcribed primarily in sporangia, chilled sporangia, zoospores, or germinated cysts, with most peaking in zoospores. About one quarter exhibit constitutive expression, and few are expressed primarily in mycelia.

Based on the number of genes having changes in relative mRNA level, several families tend to associate with one or two life-stages although there is not an absolute linkage. For example, 17 of the 34 expressed bZIP proteins have their highest mRNA levels in zoospores. The others are either expressed constitutively (nine) or show peak expression in mycelia, sporangia, or germinated cysts. This diversity of expression is consistent with prior studies based on RT-qPCR [27]. Since bZIPs act as dimers, their activity may be determined by the combination of stage-specific and constitutive subunits.

The association of protein families with life-stages becomes more apparent when summed CPM values are considered. This indicates that HLH and TUB factors associate more with the sporangial cleavage stage, while TAZ and HTH proteins peak more in zoospores. The group showing the least variation during development is the NFY family. This is unexpected since members of the family bind the CCAAT box, which is over-represented in stage-specific promoters [28]. This may signal that NFY activity is regulated mostly post-translationally, including by heterodimerization.

### Protein kinases and phosphatases

About three-quarters of genes encoding canonical protein kinases and one-third encoding protein phosphatases are expressed preferentially in one or more spore stages (Fig. 4e, f). Passing the expression threshold of CPM ≥ 1.0 were 337 of 354 genes annotated as kinases and 77 of 79 classified as phosphatases. A previous study [29] concluded that 31 kinases have defective catalytic domains, but the fractions of defective and active kinases being expressed was similar (30/31 and 303/307, respectively). The average CPM of defective kinases was slightly lower than that of active kinases, 59 versus 90.

The kinase group with the most spore-induced members is the calmodulin-regulated (CAM) family, with 40 of 69 genes showing >5-fold higher mRNA in sporangia compared to mycelium, with most induced in zoospores. Strong spore-induced patterns were observed for 15 of the 46 AGC kinases, 12 of 44 CMGC kinases, 9 of 14 STE kinases, and 44 of 129 TKL kinases. Little variation was observed for the casein kinase (CK) group. As seen in many of the prior gene categories, few kinases were expressed preferentially in germinated cysts. When expression was examined on the basis of total CPM, STE kinases showed a strong bias towards sporangia, and calmodulin-regulated kinases for cleaving sporangia.

A striking difference between kinases and phosphatases becomes evident when CPM values are examined. Although more kinases than phosphatases are expressed preferentially in spores and most phosphatases are fairly constitutive, opposite conclusions are drawn from CPM values. While total kinase CPM increases modestly in spores compared to hyphae (by 89%), protein phosphatases rise seven-fold. Interestingly, the bulk of the increase in phosphatase mRNA is due to two types of genes. One encodes Cdc14 (PITG_18578), which is known as a cell cycle regulator and in *P. infestans* is essential for sporulation. Its mRNA peaks in sporangia at a CPM of 14,061, which corresponds to more than half of total protein phosphatase CPM and 1.4% of total cellular mRNA. The other type of phosphatase comes from a cluster of four genes (such as PITG_11238) that are annotated as NLI interacting factors, which regulate elongation by RNA polymerase by dephosphosphorylating its C-terminal domain. Their expression peaks in cleaving sporangia at 0.7% of total mRNA.

### Nutrient transporters

**Fig. 5:**
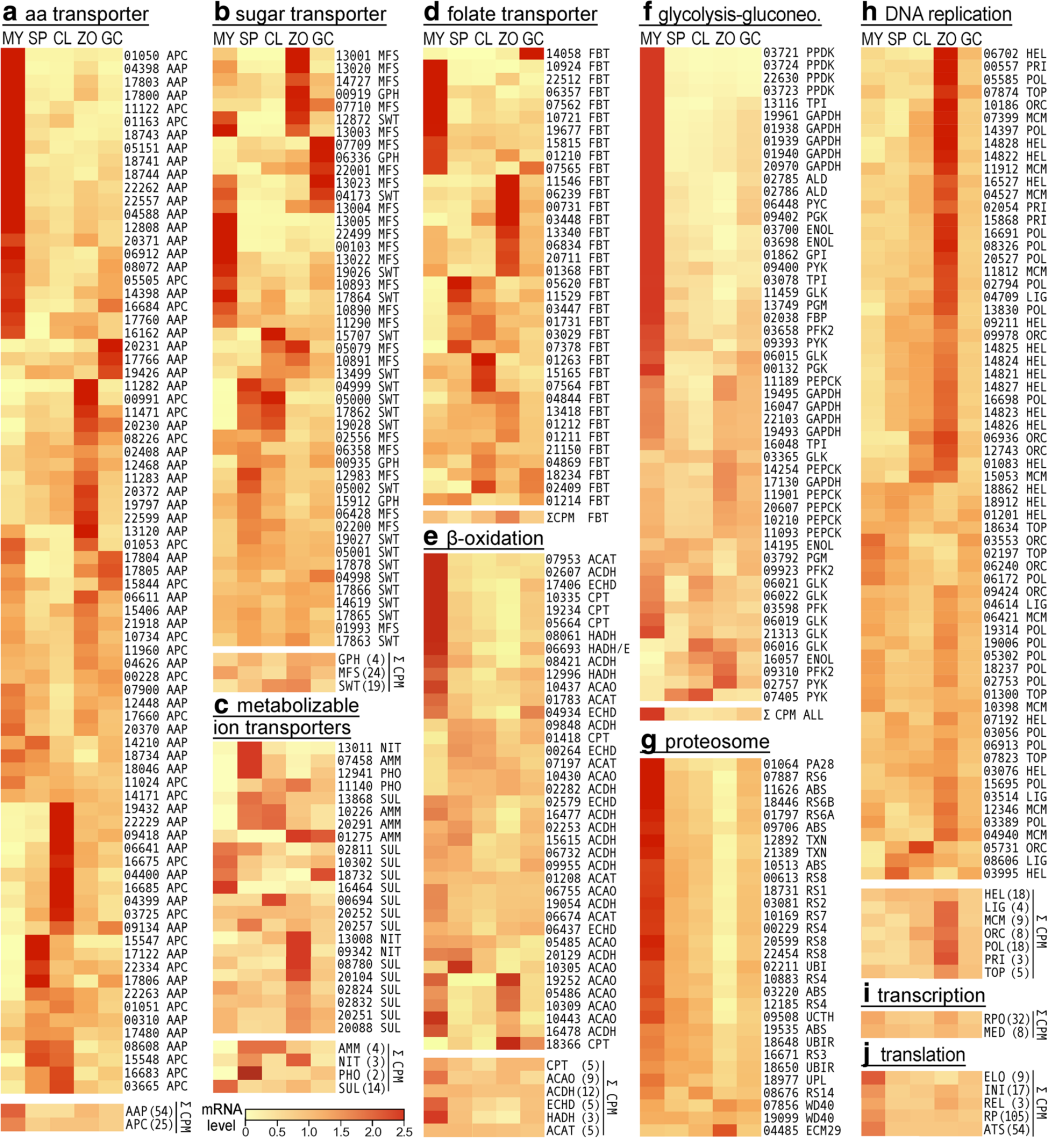
Expression patterns of selected genes involved in metabolism. The figure is formatted as in Fig. 4. Gene numbers not shown are presented in Additional file 5. **a** amino acid transporter. **b** sugar transporter. **c** metabolizable ion transporters. **d** folate transporter. **e** β-oxidation. **f** glycolysis and gluconeogenesis. **g** proteosome. **h** DNA replication. **i** transcription. **j** translation

Several metabolite transporters show dynamic patterns of expression during the life cycle (Fig. 5). These include amino acid transporters, which belong mostly in the amino acid auxin permease and amino acid-polyamine-organocation groups (AAP and APC; Fig. 5a), sugar transporters in the major facilitator, glycoside-pentoside-hexuronide, and SWEET groups (Fig. 5b), inorganic ion transporters (Fig. 5c), and folate-biopterin transporters (Fig. 5d).

About one-third of amino acid transporters are expressed mostly in hyphae, with the rest peaking in a spore stage or germinated cysts (Fig. 5a). This is one of the few categories of genes that have members that are up-regulated in the latter. Despite the many spore and germinated cyst-induced transporters, the aggregate CPMs of both AAPs and APCs are much higher in hyphae. This is logical since spores or germinated cysts are unlikely to acquire much nutrients from their external environment.

A similar contrast between expression pattern and CPM was seen for sugar transporters (Fig. 5b). Less than half are expressed constitutively, with most having the highest mRNA in hyphae, zoospores, or germinated cysts. Most SWEETs tend towards constitutive expression or greater expression in sporangia, most GPH transporters are higher in zoospores or germinated cysts, and most MFS transporters peak in zoospores or hyphae. It should be noted that this analysis may have omitted some major facilitator superfamily proteins that translocate sugars, as it was limited to those that contain the PFAM 00083 Sugar_tr domain.

Folate-biopterin transporters (FBT, Fig. 5d), which take up cofactors for methionine and purine synthesis, comprise an unusually large family in *P. infestans* compared to other taxa, with 36 expressed genes. Multiple FBTs have higher mRNA levels in hyphae and spores, but only one (PITG_14058) was expressed preferentially in germinated cysts. The highest total CPM was in zoospores, which was 69% higher than hyphae and 348% higher than sporangia. Zoospores are known to contain low levels of methionine [30], so the up-regulation of FBTs in that stage may be related to the resumption of methionine biosynthesis. The last enzyme in the pathway for methionine biosynthesis, methionine synthase (PITG_01804), increased 1.9 fold in zoospores compared to sporangia. In contrast, mRNA levels of genes encoding enzymes for purine synthesis, which also rely on folate cofactors, did not rise in zoospores. Instead, the aggregate CPM of enzymes in *de novo* biosynthesis and salvage pathways for purine were 34 and 47% lower in zoospores compared to hyphae, respectively.

### Transporters of metabolizable inorganic ions

Transporters of ammonium, nitrate, phosphate, and sulfate showed dynamic changes in expression (Fig. 5c). Diverse patterns were exhibited by the 13 sulfate transporters, which may participate in uptake for anabolism or the removal of waste. While some (*e.g.* PITG_16464) were essentially hyphae-specific, others were expressed primarily in sporangia (PITG_20257), zoospores (PITG_08780), or germinated cysts (PITG_18732). Based on CPM values, however, total sulfate transporters show a 6-fold decline in stages after hyphae.

Ammonium transporters may also be used for obtaining substrates for anabolism or waste removal. All four genes encoding these proteins were >100-fold higher in sporangia (PITG_07458, 10226, and 20291) or zoospores (PITG_01275) than hyphae. We considered whether hyphal expression might have been higher if ammonium-based media instead of the amino acid-rich rye media had been used. However, assays of hyphae grown on rye media, minimal media with amino acids, and ammonium-based minimal media indicated that mRNA levels did not increase when ammonium was the nitrogen source (data not shown).

None of the three expressed nitrate transporters showed much transcription in hyphae, instead being up-regulated in zoospores (PITG_09342 and PITG_13008) or sporangia (PITG_13011). It is notable that the latter two genes had distinct patterns of expression despite being physically linked and presumably evolved by duplication from a common ancestor. Whether zoospores use nitrate from the environment is unknown, but such genes might allow the acquisition of nitrate from soil or leaf surfaces.

### Metabolic pathways: β-oxidation

The formation of sporangia involves a transition from growth supported by nutrient absorption to a reliance on stores; sporangia remain hydrated and metabolically active. As lipids are believed to be the major carbon store [31], genes involved in β-oxidation were examined (Fig. 5e). Very few of the genes were spore-induced, with most mRNA levels either falling in spores compared to hyphae or staying constant. This was seen for the four main enzymes involved in β-oxidation (acyl-CoA oxidase/dehydrogenase, ACAO; enoyl-CoA hydratase, ECHD; hydroxy acyl-CoA dehydrogenase, HADH; acyl-coA acetyl transferase; ACAT) and carnitine palmitoyltransferase (CPT), which is needed to process long-chain fatty acids into the pathway.

It was notable that several ACAOs that had much higher mRNA levels in hyphae than sporangia regained expression in zoospores, such as PITG_19252. One CPT, PITG_18366, was also up-regulated in zoospores. However, aggregate CPM levels either remained unchanged (ACAT) or declined (HADH, ECDH, ACAO) in spores. HADH, ECDH, and CPT mRNAs began to recover expression upon cyst germination.

### Glycolysis and gluconeogenesis

We also examined glycolysis and gluconeogenesis, pathways that generate energy and metabolic intermediates (Fig. 5f). The majority of genes representing each enzymatic activity show a strong decline in mRNA in the post-hyphal stages, and this was also reflected in their total CPM. The main exception was phosphoenolpyruvate carboxykinase (PEPCK), which is part of the oxaloacetate shuttle that moves carbon towards glucose in gluconeogenesis. All five PEPCKs had slightly higher mRNA levels in zoospores than hyphae, and this is mirrored in their total CPM values.

Despite the possibility that increased gluconeogenesis might be needed to generate metabolic intermediates in spores or germinated cysts, this was not consistent with the data. Although PEPCK transcripts are maintained through each life stage, other enzymes specific to gluconeogenesis showed a major drop after hyphae. For example, the gene encoding fructose-1,6-bisphosphatase (FBP), which catalyzes a unidirectional reaction that drives carbon towards glucose, is expressed at high levels in hyphae and 4 to 10-fold lower levels in spores, although its mRNA level starts to recover in germinated cysts. A similar pattern was seen for pyruvate carboxylase (PYC), another unidirectional enzyme which moves carbon from pyruvate towards glucose. Although not shown in the figure, a similar pattern of high mRNA in hyphae and low mRNA elsewhere was observed for the two malate dehydrogenases, PITG_13614 and PITG_15476, which act after PYC to move carbon into gluconeogenesis using the oxaloacetate shuttle.

As in β-oxidation, most genes that had reduced mRNA levels in sporangia compared to hyphae started to regain expression in germinated cysts. However, unlike β-oxidation, genes encoding the same activity tended to display similar patterns of expression. Likely contributing to this is the fact that many are encoded by physically linked genes evolved from common coding and promoter sequences. For example, aldolase (ALD) is encoded by two linked genes, PITG_02785 and PITG_02786.

Exceptions to the pattern of co-regulation within families are several enzymes in the ATP-generating pay-off phase of glycolysis. For example, while enolases (ENOL) PITG_03698 and PITG_03700 have high mRNA in hyphae but low levels in the other stages, enolase PITG_14195 exhibited constitutive expression. An explanation for the difference may be that the enzymes may play distinct roles. PITG_03698 and PITG_03700 encode conventional cytosolic enzymes while PITG_14195 protein is predicted to reside in mitochondria, which is an atypical location. Mitochondrial forms of glycolytic enzymes have been reported in other stramenopiles [32].

A second exception to coordinate expression within a family are the seven genes encoding glucokinase, GLK. Five have the highest mRNA levels in hyphae, but one (PITG_06016) contrarily has eight-fold higher expression in zoospores than hyphae; its CPM in zoospores is five times greater than the other genes combined. This occurs even though PITG_06016 is part of a cluster with four other GLK genes. An additional but unlinked GLK, PITG_03365, shows minor changes between life stages and represents 5 to 18% of total GLK CPM at each stage.

Diversity was also seen within the four expressed pyruvate kinases (PYKs). Two linked genes (PITG_09393, PITG_09400) have peak mRNA levels in hyphae, five-fold more than in sporangia, while two unlinked genes have five to ten-fold higher mRNA in cleaving sporangia or zoospores than hyphae (PITG_07405, PITG_02757). Total PYK CPM is higher in hyphae than the other life stages since PITG_09400 is expressed eight-fold higher than the other genes.

Our analysis of the PYKs also revealed an instance of diversity between isolates 1306 and 88069. In 1306, PITG_09394 showed a CPM below 0.01 even though the neighboring PYK gene PITG_09393 was expressed up to 347 CPM. PITG_09394 is expressed in isolate 88069 (CPM = 35), suggesting that the gene in 1306 is either a pseudogene, diverged to the point that RNA-seq reads will not map, or absent. This situation was not common with metabolic enzymes, but was observed frequently for transporters, which more often exist as multigene families. For example, sulfate transporter PITG_20090 had virtually no mapped reads in isolate 1306, but was expressed in every developmental stage of 88069.

### Proteosome activity and translation

We also examined the proteosome pathway, which might be involved in recycling proteins during growth and developmental transitions, or helping spores adapt to nutrient limitation as in yeast [33]. This involved measuring the expression of its structural, catalytic, and regulatory subunits (Fig. 5g). mRNA levels of nearly all genes decline dramatically in spores, and start to recover upon cyst germination. This trend is reminiscent of many genes in glycolysis and β-oxidation. The data also suggest that protein turnover is a not a major source of nutrients for spores, and may explain why mRNAs of core metabolic enzymes fall after sporulation: not only are energy and other metabolic needs reduced, less *de novo* synthesis to replace enzymes may be required.

A diminished need for new proteins may also explain why most genes involved in translation show reduced mRNA levels in spores. Translation initiation factors, elongation factors, release factors, ribosomal proteins, and tRNA charging enzymes fell by 45–61% in sporangia compared to hyphae (Fig. 5i). These values do not include PITG_03712, which we have annotated as elongation factor 3. This protein has been known as a fungal-specific factor that helps clear deacyl-tRNAs from the ribosome [34]. A partial sequence obtained based on a *P. infestans* protein spot from a 2-D gel was previously shown to have homology to EF-3 [35]. Based on the full-length gene sequence, we concur that *P. infestans* has a EF-3-like protein. This was not included in the tally of elongation factor CPMs since its function in oomycetes is not yet confirmed. Also, it is expressed at extremely high levels that dwarf the combined CPM of the 26 other elongation factor genes. The CPM of PITG_03712 in mycelia, sporangia, cleaving sporangia, zoospores, and germinating cysts was 3049, 33066, 22114, 8557, and 4334, respectively. Its CPM in sporangia is higher than any other gene in any life stage.

### DNA replication

Sporangia enter a period of mitotic dormancy that ends soon after cysts germinate [15, 36]. In some non-oomycetes, the transcription of genes that regulate or catalyze DNA replication is known to be reduced [37]. However, this was not the case in *P. infestans* sporangia or zoospores*.* Most genes encoding primase, DNA polymerase subunits, topoisomerase, DNA helicase, or MCM and ORC proteins (which help polymerases assemble at replication forks and origins;[38]) have similar mRNA levels in hyphae and sporangia (Fig. 5h). There were only a few exceptions to this pattern, such as PITG_12346 which encodes DNA replication licensing factor MCM3, and ORC proteins PITG_03553 and PITG_06240, showed a modest 50% decline in mRNA in sporangia compared to hyphae.

In dramatic contrast to mRNAs associated with glycolysis, β-oxidation, and the proteosome which are lowest in zoospores, the opposite trend was observed with DNA replication. Two thirds of the latter genes have peak mRNA in zoospores, >5-fold higher than in actively growing, nonsporulating hyphae. Examples are the three DNA primases (*e.g.* PITG_00557), most MCM and ORC proteins (*e.g.* PITG_07399, MCM2), and DNA polymerase subunits such as PITG_14397 (DNA pol α), which initiates replication on leading and lagging strands. Except for helicases, the trend of higher mRNA levels in zoospores persisted when analyzed on the basis of aggregate CPM values. Apparently the developmental program anticipates the need to resume replication after encystment.

### Other metabolic pathways

**Fig. 6:**
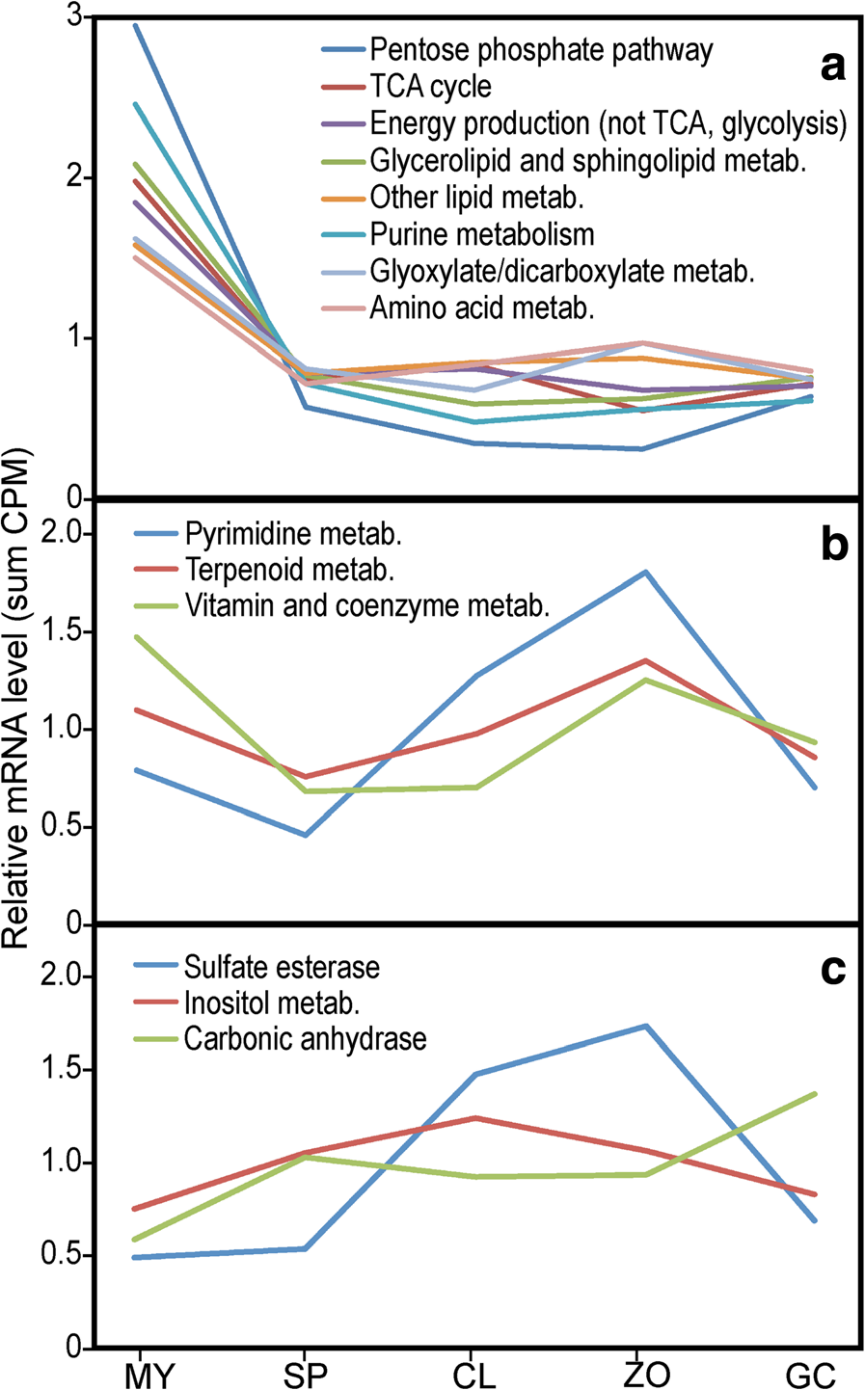
Expression patterns of additional metabolic pathways. Presented in panel **a** are the pentose phosphate pathway, TCA cycle, other genes involved in energy production, glycerolipid and sphingolipid metabolism, other lipid metabolism pathways, purine metabolism, glyoxylate and dicarboxylate metabolism, and amino acid metabolism. Shown in panel **b** are pathways of pyrimidine metabolism, terpenoid metabolism, and vitamin/coenzyme metabolism. ﻿ Panel **c** shows sulfate esterases, inositol metabolism enzymes, and carbonic anhydrases. Shown are the summed CPM values of genes in each category. The gene numbers are provided in Additional file 5

A survey of other pathways revealed trends consistent with those described above. To save space, their aggregate CPM values during development are summarized in Fig. 6. Most show >5-fold decreases in each spore stage compared to hyphae. Examples are the pentose phosphate pathway, TCA cycle, and pathways involving amino acids, glycerolipids, and purines (Fig. 6a).

A few pathways show modest two-fold declines in sporangia, with mRNA levels that recover in chilled sporangia and zoospores (Fig. 6b). These include pathways for making pyrimidines, terpenoids, and vitamins or coenzymes such as biotin, folate, riboflavin, and pantothenic acid. It is notable that folate transporters exhibited a similar pattern (Fig. 5d).

Only a few pathways exhibit more transcripts in spores (Fig. 6c). These include sulfate esterases, which act on sulfated glycosaminoglycans and glycolipids, proteins involved in inositol metabolism, and carbonic anhydrases. While the latter is best-known for its role in removing CO_2_ from within animal tissues, in *P. infestans* it may play a role in buffering pH or producing oxalacetate in concert with PEP carboxylase [39].

### Plant cell wall degrading enzymes (CWDEs)

Oomycetes express many enzymes capable of degrading cell walls. Both plant and oomycete cell walls are mainly cellulosic, so we focused on the expression of those that degrade components believed to be plant-specific [40]. These include members of carbohydrate esterase family CE5 (cutinase), carbohydrate esterase family CE8 (pectin esterase and methylesterase), pectate lyase families PL1, PL2, and PL3, and glycosyl hydrolase families GH28 (polygalacturonase), GH53 (endo-β-1,4-galactanase), GH54 (β-xylosidase), GH78 (α-L-rhamnosidase), and GH105 (rhamnogalacturonyl hydrolase).

**Fig. 7:**
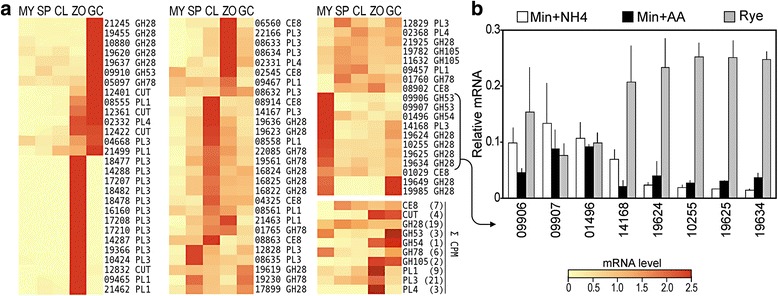
Expression patterns of genes encoding plant cell wall degrading enzymes. **a** heatmaps formatted as in Fig. 4. **b** expression of eight selected CWDE genes in ammonium sulfate or amino acid-based minimal media (Min + NH4, Min + AA), or rye media. Enzyme functions associated with each category are described in the main text

Only 11% of CWDE genes display significant expression in hyphae (Fig. 7a). The vast majority are instead up-regulated in chilled sporangia and zoospores, with a few up-regulated in germinated cysts or sporangia. The timing of expression of each enzyme has been suggested to reflect the stage of infection at which they are needed to breach the host cell wall [41]. Although most families contained a few members that were expressed at similar levels in all stages, there was a trend for GH78 and CE8 genes to be induced in cleaving spores, PL and CE5 in zoospores, and GH28, GH53, and GH78 in germinated cysts. A similar order of expression is seen when summed CPM values are considered, except for GH28. Even though most GH28 genes peaked in germinated cysts, two highly-expressed genes (PITG_17899, 19619) were more constitutive and several were expressed at their highest levels in rye media, such as PITG_19624.

The high level of expression shown by several CWDE genes in rye media was shown to be attributable to induction by plant compounds. This involved comparing their expression in rye media with ammonium or amino acid-based minimal media (Fig. 7b). Of eight genes tested, five are expressed at high levels only in rye media. Two examples are polygalacturonase PITG_19634 and pectate lyase PITG_14168, which are induced 10-fold in rye compared to minimal media. In contrast, genes such as PITG_09906 were expressed similarly in all media. We conclude that some CDWEs are regulated by their substrate, and the rest hard-wired into the developmental program of *P. infestans.* CWDEs in fungi are known to be regulated by several factors including the presence of a plant, nutrient levels, and pH [42, 43].

### Other genes related to pathogenicity

**Fig. 8:**
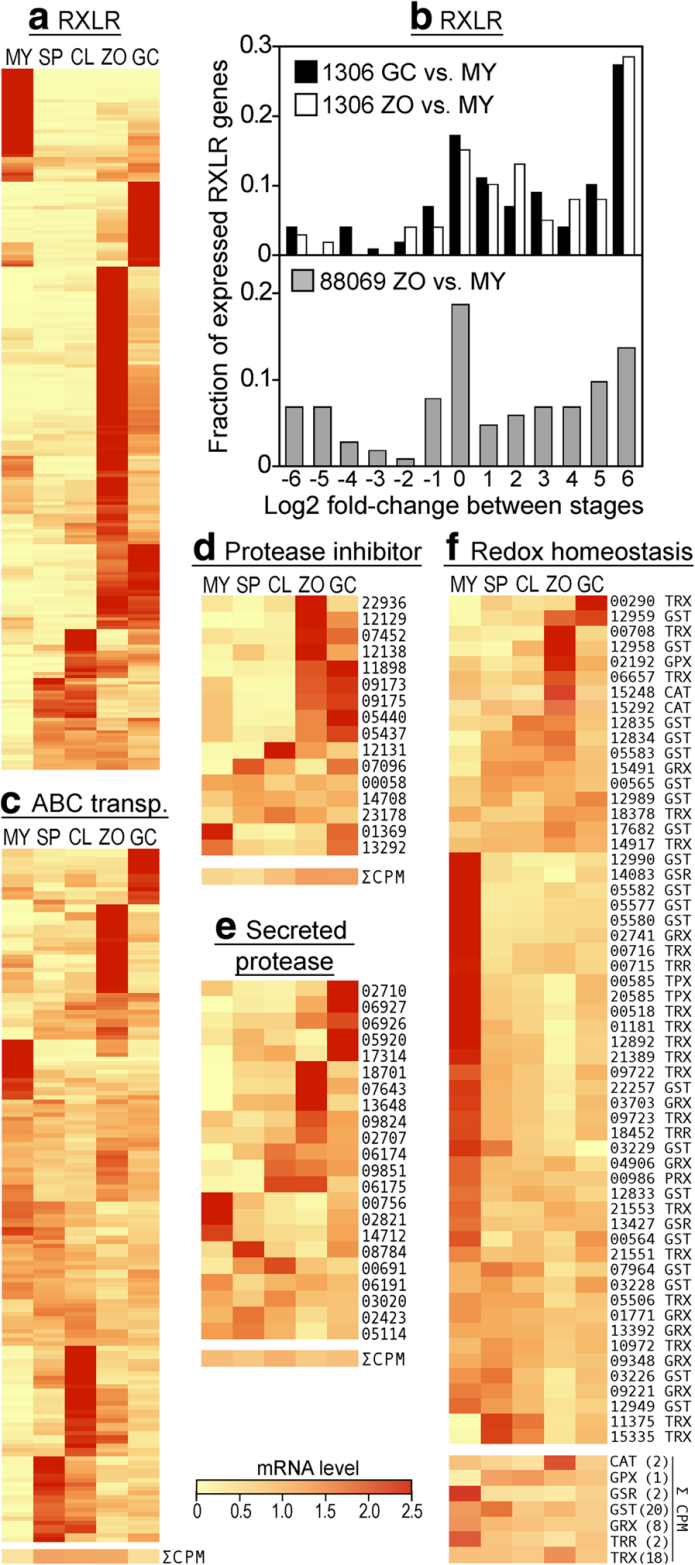
Expression patterns of genes potentially playing roles in pathogenesis. **a** expression of RXLR genes in isolate 1306. **b** comparison of RXLR expression in 1306 and 88089. Also shown are the expression patterns of **c** ABC transporters; **d** secreted protease inhibitors; **e** secreted proteases; and **f** genes involved in redox homeostasis

RXLR effectors modulate host physiology during plant infection. Studies in *P. infestans* and relatives have shown that many are up-regulated in zoospores or germinated cysts [44], and we quantified this genome-wide in both 1306 and 88069 (Fig. 8a, b). In 1306, 85% of predicted RXLR genes are induced in sporangia, zoospores, or germinated cysts, with most being up-regulated in zoospores. Similar results were observed in 88069.

Secreted proteases and protease inhibitors also contribute to pathogenicity. Like RXLRs, most are induced in zoospores or germinated cysts (Fig. 8d, e). The aggregate CPM of protease inhibitors also peaks in zoospores and germinated cysts. In contrast, although most genes encoding secreted proteases are induced in those life stages, their combined CPM is constant across all stages. This is because the spore-induced genes are counterbalanced by genes such as PITG_00756, a highly-expressed carboxypeptidase that is transcribed more in hyphae.

ABC transporters are thought to play roles in pathogenicity due to their potential role in phytoalexin efflux, and they also contribute to fungicide resistance [45, 46]. About two-thirds of this large family of genes showed dynamic variation in mRNA in the different life stages (Fig. 8c). A majority were induced in sporangia, cleaving sporangia, or zoospores. The collective CPM of all ABC transporters was also highest in these stages.

Since plants form reactive oxygen as part of their defense responses, genes that may contribute to redox homeostasis were examined (Fig. 8f). These include catalases and proteins in the glutathione and thioredoxin cycles. About half were expressed primarily in hyphae, and one-quarter in spores or germinated cysts. Both catalase genes (PITG_15248 and PITG_15292) were up-regulated in zoospores.

A disproportionate number of genes predicted to encode secreted pathogenicity factors lacked mapped reads in isolates 1306 and 88069. While 90% of total genes had CPM > 1.0, this was only the case for 16 of 26 protease inhibitors, 18 of 28 NPP1 proteins, 2 of 4 berberine bridge proteins, and 258 of the 551 RXLR proteins predicted from the *P. infestans* reference genome. While expression might occur in growth stages not examined in this study, many of these exist as gene families in which selection against pseudogenization would be relaxed. Many of these have also been shown to reside in hypervariable parts of the genome [44]. In contrast, 21 of the 22 genes annotated as secreted proteases were expressed.

### Exocytosis and vesicle transport

Since the secretion of effectors is an important component of pathogenesis [44], we examined genes involved in protein export. *P. infestans* orthologs of yeast genes that traffic secreted proteins from the endoplasmic reticulum to Golgi (SAR1, SEC13, SEC17, SEC18, SEC19, SEC22, SEC23, SEC24; [47]) each displayed only minor changes between life stages, ≤2-fold. Orthologs of yeast genes involved in secretion from the Golgi (SEC1, SEC4, SEC7, SEC10, SEC14) showed more variation; while their summed CPM was fairly constant, SEC1 ortholog PITG_15867 was induced 70-fold in sporangia compared to hyphae, and then declined in subsequent stages. This protein binds to SNARE complexes to stimulate membrane fusion and exocytosis [48]. A similar pattern was observed for SEC10 ortholog PITG_18158.

Interestingly, all 14 expressed *P. infestans* genes annotated as encoding SNARE proteins (having PFAM domain PF05739) were transcribed at higher levels in sporangia, cleaving sporangia, or zoospores compared to hyphae. The most extreme was PITG_10966, which had 100-fold higher mRNA in sporangia than hyphae. In contrast, diverse patterns were exhibited by genes encoding Rab GTPases, which were either expressed at similar levels in all stages (19 genes) or were higher in hyphae (eight), sporangia (nine), cleaving sporangia (four), or zoospores (six). Such diverse patterns are not surprising since Rab GTPases affect many steps of membrane traffic other than secretion [49].

The induction in spores of the genes discussed above may reflect the need for secretory processes in germinated cysts to release effectors for plant infection. Alternatively, they may act to secrete molecules for growing the *P. infestans* cell wall in germ tubes. The genes may also participate in zoosporogenesis or encystment, which involves a massive reorganization of vesicles to assemble the zoospore plasma membrane or the cyst wall [6].

### Inhibitors of calcium signaling affect transcription

**Fig. 9:**
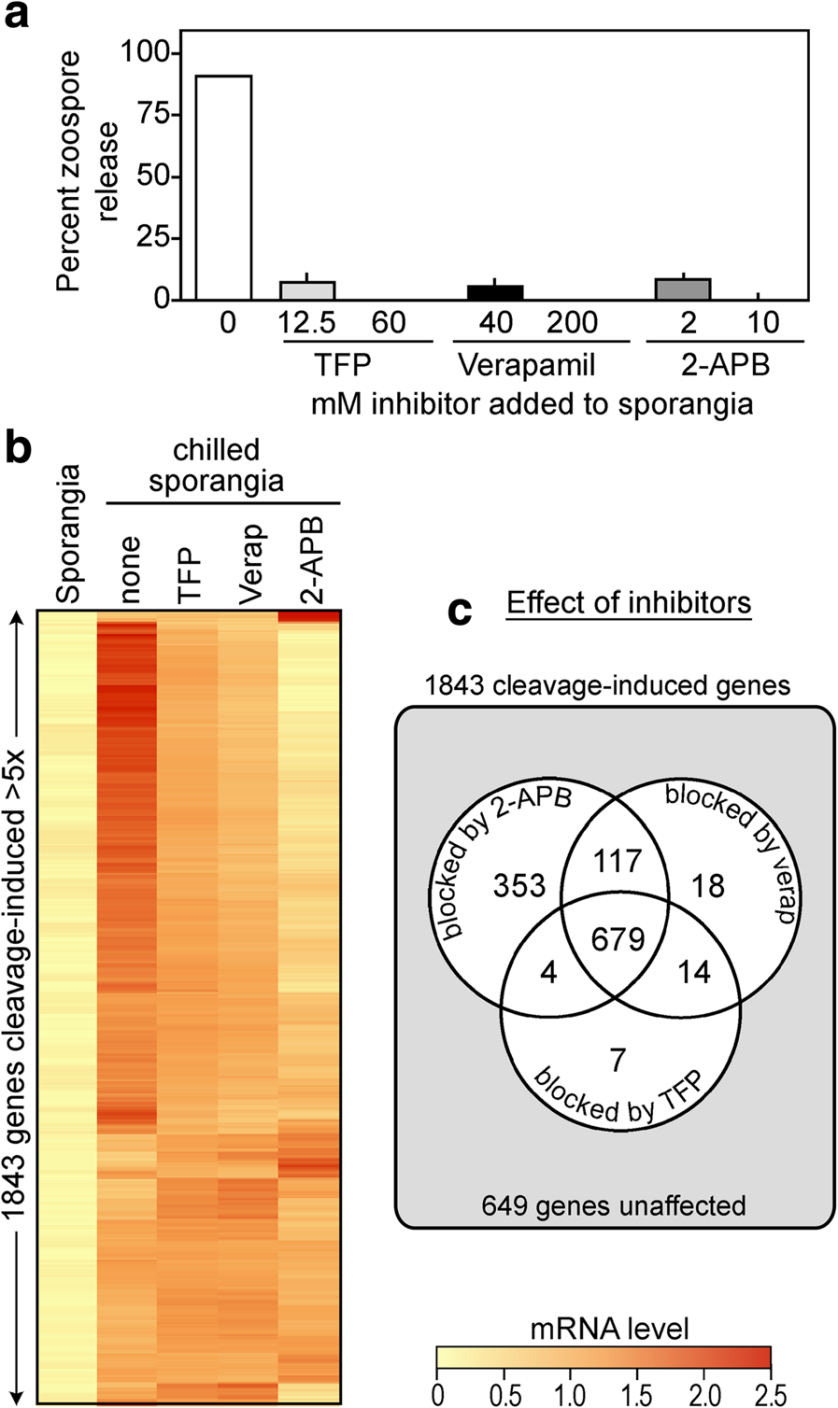
Effects of inhibitors of calcium signaling. **a** effect of trifluoroperazine (TFP), verapamil, or 2-APB on zoospore release from sporangia of isolate 88069. **b** Heatmap showing expression of 1843 cleavage-induced genes in presence and absence of inhibitors. **c** Comparison of effects of the three inhibitors on the induction of the 1843 genes

The role of calcium signaling was examined in more detail due to the dynamic changes described above for calcium-binding proteins and calcium channels. We previously showed that 2-aminoethoxydiphenyl borate (2-APB), verapamil, and trifluoroperazine block zoospore release in isolate 1306 [50]. Figure 9a shows that these have similar effects on isolate 88069. Verapamil inhibits voltage-gated calcium channels, trifluoroperazine is a calmodulin antagonist, and 2-APB inhibits the inositol 1,4,5-trisphosphate (IP_3_)-induced release of calcium from stores, although the latter may also effect some other calcium channels [51, 52].

To understand how calcium-related signaling affects gene expression during zoosporogenesis, we added the three inhibitors (or solvent alone) to sporangia, chilled the sporangia to initiate zoosporogenesis, and then after 30 min extracted RNA which was subjected to RNA-seq analysis (Fig. 9b,c). These samples were prepared in parallel with the 88069 material described in the 2008 study, but were not analyzed previously. Of 1843 genes classified as cleavage-induced in the absence of inhibitor based on a five-fold change threshold, one or more of the three inhibitors blocked the normal induction of 1192 genes (FDR <0.05). The genes affected by verapamil and trifluoroperazine were nearly identical, suggesting that calcium introduced by voltage-gated channels acts mainly through calmodulin or related proteins. About 353 additional genes were inhibited by 2-APB, which suggests that the calcium release blocked by 2-APB is mediated through proteins distinct from those affected by verapamil, or that it was more potent at the concentration tested.

The expression of 649 cleavage-induced genes was unaffected by any inhibitor. This is consistent with the premise that both calcium-dependent and independent signals are active during zoosporogenesis [50]. We previously suggested that the latter might be regulated by IP_3_ or diacyl glycerol produced by phospholipase C, PLC. While *Phytophthora* apparently does not encode a canonical PLC, a novel protein with PLC activity has been discovered recently [53].

### Studies of a sporulation-induced protein kinase

Since protein kinases were highly overrepresented in genes induced in both sporangia and cleaving sporangia, we chose one for further analysis. PITG_10884 encodes a 540-aa AGC kinase (PKS1) that is induced 48-fold in sporangia compared to hyphae, and bears a PH protein interaction domain at residues 43-121 and a kinase domain at residues 141–368.

**Fig. 10:**
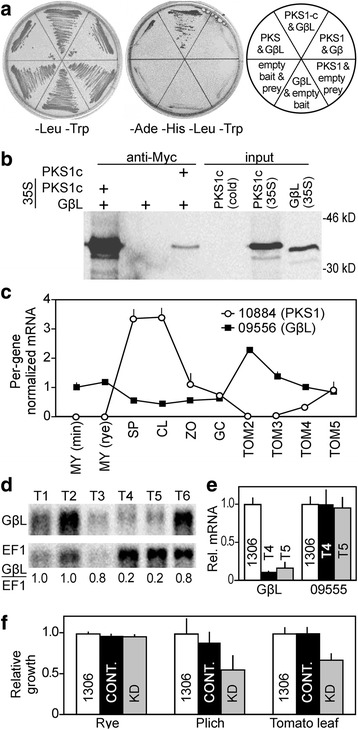
Studies of sporulation-induced protein kinase and and interactor Gβ-like protein. **a** Two-hybrid analysis showing tests of interaction between full-length kinase (PKS1), kinase catalytic domain (PKS1c; amino acids 135 to 466), Gβ-like protein (GβL), and canonical Gβ. An interaction between PKS1c and GβL is indicated by the strong growth in the center upper panel of the right culture plate. **b** Co-immunoprecipitation of GβL with PKS1c, showing input and co-precipitated amounts. **c** Expression patterns of *PKS1* and *G*βL in mycelia grown on minimal media containing casamino acids (MY min) or rye media (MY rye), sporangia (SP), sporangia undergoing zoosporogenesis (CL), zoospores (ZO), germinated cysts (GC), and tomato leaves at 2 to 5 dpi (Tom). **d** Northern blot of selected transformants containing GβL silencing construct, using the gene for elongation factor 1-α (EF1) as a loading control. The ratio of GβL and EF1 relative to wild type is shown below the blots. Transformants T1, T2, and T6 show levels of GβL equivalent to wild-type, while T4 and T5 are knocked-down by four to five-fold. **e** RT-qPCR of wild-type strain 1306 with T4 and T5, using primers for GβL (PITG_09556) and flanking gene PITG_09555, which encodes a predicted ubiquitin-ribosomal protein fusion. This confirms the knockdown and indicates that flanking genes are not co-silenced. **f** Relative growth of 1306, control transformants, and T4 and T5 knockdown (KD) transformants on rye media (rich media), Plich media (semidefined), and *in planta.* The control transformants (CONT) are the average of non-silenced transformant T1 and two strains expressing the GUS transgene. Growth on media was determined on a dry weight basis, while growth in tomato was determined by qPCR using primers for a high-copy DNA locus [72]. Values are adjusted to 1306 equals 1.0

Yeast two-hybrid was used to identify proteins that interact with the kinase. A full-length PKS1 bait was moderately toxic to yeast, but normal growth occurred when the bait contained only the catalytic domain (PKS1c). Using this against a prey library made from cDNA of sporulating mycelia, we identified an interacting Gβ-like protein produced from PITG_09556 (GβL). The latter is a member of an evolutionarily conserved group that is distinguished from classic Gβ proteins by an expanded C-terminal domain [54]. GβL proteins are not Gβ mimics, although both contain multiple WD40 domains, which mediate protein-protein interactions. Expression of PKS1c bait with full-length GβL prey activated the reporters, allowing growth on quadruple drop-out media (Fig. 10a). Some growth was also observed when full-length PKS1 bait was expressed with Gβ-like prey. No interaction was observed between PKS1 and the canonical Gβ protein of *P. infestans.*

We confirmed the interaction between PKS1 and GβL by co-immunoprecipitation (Fig. 10b). This employed ^35^S-labeled, *myc*-tagged PKS1c and GβL made by in vitro transcription and translation. Anti-*myc* precipitated GβL in the presence but not absence of *myc*-PKS1c.

The relative expression patterns of the *PKS1* and *GβL* genes are shown in Fig. 10c. While the kinase gene shows little expression in mycelia, it is expressed at high levels in sporangia and cleaving sporangia, and increases in late stages of tomato infection when sporulation is occurring. In contrast, *GβL* is expressed in all life stages, declining by about 50% in the spore stages relative to hyphae and increasing during early infection timepoints.

We next attempted to silence the *PKS1* and *GβL* genes by DNA-directed RNAi. Two transformants (T4 and T5) down-regulated by ~80% were obtained for *GβL* based on Northern blot analysis (Fig. 10d). However, none silenced for *PKS1* were obtained in over 200 transformants obtained with sense, antisense, or hairpin constructs. Despite the fact that *PKS1* is expressed primarily in sporangia, it is possible that silencing is lethal.

RT-qPCR using new mRNA preparations confirmed the knockdown of *GβL*, indicating that the gene was expressed at 10 and 16% of wild-type levels in T4 and T5, respectively (Fig. 10e). We also tested the genes flanking *PKS1* to confirm that the heterochromatinization phenomena associated with knock-downs in *P. infestans* had not spread to adjoining genes [55]. Gene PITG_09555, which is located immediately to the left of *PKS1* in the genome assembly, was expressed at similar levels in wild-type, T4, and T5 (Fig. 10e). We could not obtain data for the gene to the right of *PKS1,* PITG_09557, by RT-qPCR since Ct values using RNA from hyphae were similar to the no-template control, even in wild-type. This was not surprising since no reads mapped to this predicted gene in RNA-seq. The next gene is 52 kb to the right, and was not tested. Our prior studies indicate that heterochromatinization associated with silencing can spread several hundred nt [55].

Phenotypic analysis of the two *GβL*-knockdown strains indicated that silencing did not have a significant effect on sporulation, growth on rich media, zoosporogenesis, cyst germination, or appressorium development based on comparisons with the wild-type progenitor strain 1306 or three control transformants (Additional file 4). There was also no obvious difference in swimming behavior. However, growth was reduced by about one-third on the less-rich Plich media and *in planta,* based on measurements of *P. infestans* DNA by qPCR (Fig. 10f) and the number of sporangia formed per leaflet (Additional file 4). In other eukaryotes, Gβ-like proteins play roles in multiple aspects of growth and development by helping to assemble signaling protein complexes [54, 56]. While GβL may play a role in *P. infestans* spores through interactions with PKS1, the phenotypic effects of the knock-down on hyphal growth may be due to its other interactions.

## Conclusions

Our earlier finding [3] that the *P. infestans* transcriptome shifts dramatically during the life cycle has been reinforced through the use of RNA-seq. The new data are more comprehensive since greater than twice the number of genes were measurable than before, and more informative due to better annotations. For example, the number of genes tagged as being involved in DNA replication rose from 20 to 65, ion channels increased from 32 to 67, and plant wall-specific CWDEs went from 7 to 75. The new data should also be more accurate since Affymetrix microarrays have a limited dynamic range. A whole-transcriptome Nimblegen array has been used with some of the life stages examined here, but we have found that the high background of that platform precludes the study of genes with below-average expression [9]. The robustness of our new dataset is also enhanced by our use of two isolates of *P. infestans.*

Increased clarity has also resulted from our choice to consider summed CPM values when interpreting the data. Our prior study found it challenging to draw conclusions about the expression of many cellular pathways since gene families often included up-regulated, down-regulated, and constitutive paralogs*.* CPM analysis indicated more clearly that most metabolic pathways were down-regulated in spores. This assumes that a weakly transcribed paralog contributes less than paralogs with higher CPM, which may not always be true.

Whether protein and RNA levels change in parallel should be considered. Sporangia can live for several days, during which time considerable protein turnover and *de novo* translation may occur [57]. Smaller protein shifts may occur in the briefer zoosporogenesis and zoospore stages, where chilling may impair translation [58], although a two-dimensional gel study did identify some protein changes [59]. While several higher-throughput proteomics studies have examined life stages of two species of *Phytophthora* [60, 61], we find only partial overlap between the changes in those studies and ours, although disparities may be attributable to differences between species or sampling parameters. Weak associations between mRNA and protein abundance are common in many systems [10]. Correlations between our results and transcriptional shifts in *Phytophthora capsici* were also only moderate, perhaps since our studies also used different conditions [62].

Even if there is a disconnect between mRNA and protein levels, the timing of mRNA induction may telegraph when gene products are needed. For example, genes induced in sporangia and zoospores may encode proteins needed for early and late stages of host penetration, respectively. That calcium inhibitors affect only a subset of cleavage-induced genes is evidence that genes are induced by multiple pathways, and possibly at different times which could be identified through more detailed timecourses.

Even if the number of new proteins made in a transient stage such as cleaving sporangia is limited, low levels of new proteins may be enough to affect development. For example, a few molecules of PKS1 may be sufficient to modify GβL. That the sporangia-induced PKS1 binds the constitutive GβL is reminiscent of our prior finding that a zoosporogenesis-induced kinase binds a constitutive transcription factor [63]. The results suggest a paradigm where cellular regulation in spores is driven by induced proteins that modify existing machinery.

## Methods

### *P. infestans* growth and development

Strains 1306 (A1 mating type, isolated from tomato, California USA) and 88069 (A1, potato, the Netherlands) were maintained in the dark in rye-sucrose agar [64] at 18 °C. Developmental stages were obtained as described from rye media cultures [3]. In brief, nonsporulating mycelia were obtained from 3-day cultures. Sporangia were scraped from the surface of 10-d cultures in water, purified by nylon mesh filtration, and either pelleted by centrifugation and frozen pending RNA extraction, or chilled at 10 °C for 60 min to initiate zoosporogenesis. Zoospores were obtained by incubating the sporangia for an additional 90 min, pelleted in a centrifuge, and frozen pending RNA extraction; about half may have encysted when being concentrated by centrifugation prior to freezing. Germinating cysts were obtained by adding CaCl_2_ to 0.25 mM, vortexing, and incubating the resulting cysts for 6 h in water at 18 °C. As described in Results, some experiments used minimal medium based on the recipe of Xu [65], the latter with (NH_4_)_2_SO_4_ replaced by 1% casamino acids, or Plich semidefined media [66]. Some experiments also chilled sporangia at 4 °C in the presence of 10 μM 2-APB, 60 μM trifluoroperazine, or 200 μM verapamil for 3 h to measure their effect on zoospore release, or 1 h to obtain RNA for RNA-seq.

Plant infections used tomato *cv.* Moneymaker (for pathogenicity assays) or New Yorker (for gene expression analysis). Plants were grown with a 12 h light/dark cycle (25 °C day, 350 μmol · m^-2^ · s^-1^ fluorescent light; 18 °C night) for 4–5 weeks, and then leaves were detached, placed on 0.8% water agar, inoculated with zoospores, and incubated at 16 °C with a 12 h light/dark cycle with 115 μmol · m^-2^ · s^-1^ illumination in a clear plastic bag to maintain high humidity.

### Transformation of *P. infestans*

These were performed using the protoplast method [67]. Silencing vectors in initial experiments expressed the open reading frame of *PKS1* or *GbL1* behind the *ham34* promoter in pTEP3, which confers G418 resistance. Experiments with hairpin constructs of *PKS1* used pTOR [68]. Knocked-down strains were characterized by blot analysis using ^32^P-labelled probes and RT-qPCR, and confirmed with a minimum of three biological replicates. For RT-qPCR, cDNA was synthesized using the Maxima First-Strand RT-PCR kit (Thermo). Amplifications were performed using the Biorad CFX Connect system using the Dynamo SYBR Green qPCR kit (Thermo) with the following program: 95 °C for 15 min, followed by 40 cycles of 94 °C for 30 s, 50 °C for 30 s, and 72 °C for 30 s. Melt curves were generated to evaluate the fidelity of amplification. Expression levels were calculated using the ΔΔC_T_ method, using a constitutive gene (ribosomal protein S3a, PITG_11766) as a control [3, 69].

### RNA-seq

RNA was isolated using the Sigma Plant Mini kit. RNA-seq was performed using indexed libraries prepared using the Illumina Truseq kit, and sequenced to produce 75-nt single-end reads. These were aligned and mapped to *P. infestans* gene models using Bowtie version 2.2.5 and Tophat version 2.0.14, allowing for 1 mismatch. The number of reads and biological replicates per isolate ranged from two and four, or between four and six per developmental stage, as shown in Additional file 1. Some gene models were modified prior to analysis to correct errors, including merging some models. Expression and differential expression calls were made with edgeR using TMM normalization, a generalized linear model, and false discovery rate calculations based on the Benjamini-Hochberg method [70]. Data were trimmed to exclude unreliably-expressed genes using a CPM threshold of 1.0. Hierarchical clustering, heatmap generation, and PCA analysis were performed using Partek Genomics Suite. Gene annotations including GO terms were obtained by searching the Panther and UniProtKB/Swiss-Prot databases, supplemented by annotations of secreted pathogenicity proteins from Raffaele et al [44]. GO term enrichment analysis was performed using GOHyperGAll [71].

### Yeast two-hybrid screening

A *P. infestans* cDNA library (prey) was used to construct a GAL4 activation domain (AD) fusion library using the Matchmaker library construction and screening kit (Clontech), using the pGADT7 vector and *Saccharomyces cerevisiae* strain AH109. For the bait, a DNA fragment for the catalytic domain of *PKS1* was amplified by PCR and introduced in pGBKT7 as a fusion protein with the GAL4 DNA-binding domain (DNA-BD). After coculturing cells of strain Y187 (bait) and AH109 (prey) for 24 h at 30 °C, the mating mixture was spread on quadruple dropout plates (synthetic defined media/-leucine/-tryptophan/-histidine/-adenine/X-α-gal) for selection. Interactions were retested by co-transforming plasmids for the prey and bait into AH109.

### Co-immunoprecipitations

pGBKT7 and pGADT7, containing the catalytic domain of *Pipks1* and full length *Pigbl1*, respectively, were used as templates for in vitro transcription and translation reactions, using ^35^S-methionine with the TNT transcription/translation system (Promega). This produced the *c-Myc*-tagged catalytic domain of PKS1 and HA-tagged GβL. Co-immunoprecipitation was performed using the Matchmaker Co-IP kit (Clontech). The proteins were incubated at room temperature for 1 h, then for 1 h with c-Myc antibody, and then for 1 h with Protein A beads. After pelleting and elution of bound proteins, protein bands were identified by 10% sodium dodecyl sulfate-polyacrylamide gel electrophoresis followed by phosphorimager analysis.

## Additional files

Additional file 1: Libraries used for RNA-seq. (DOCX 64 kb)
Additional file 2: CPM values from RNA-seq of samples from isolates 1306 and 88069. (XLSX 5807 kb)
Additional file 3: Fold-change ratios and Bonferroni-Hochberg false discovery rate (FDR) values for RNA-seq data. (XLSX 15732 kb)
Additional file 4: Phenotypic characterisation of PKS1 knockdowns. (DOCX 92 kb)
Additional file 5: Gene numbers for functional categories not shown Figs. 4, 6, and 8. (XLSX 72 kb)

## Abbreviations

AAP: Amino acid auxin permease transporter
ACAO: Acyl-CoA oxidase
ACAT: Acyl-coA acetyl transferase
ACDH: Acyl-CoA dehydrogenase
ADC: Adenylate cyclase catalytic subunit
ALD: Fructose-bisphosphate aldolase
AMM: Ammonium transporter
AP2: Activating protein 2
APC: Amino acid-polyamine-organocation transporter
ATS: Amino acid-tRNA synthetase
BZP: Basic leucine zipper
CAB: Mo25/CAB39 family
CAC: Calcium channel
CAD: Cadherin
CAM: Calmodulin
CAN: Calcium-regulated nucleotidase
CAP: Calcineurin
CAP: Calcium-regulated protein phosphatase (calcineurin)
CAR: Calreticulin
CAT: Catalase
CDK: Calcium-regulated protein kinase
CEN: Centrin
CH2: C2H2 transcription factor
CK: Casein kinase
CMG: CMGC protein kinase
CMK: Calmodulin-regulated protein kinase
CNG: Cyclic nucleotide gated ion channel
CPM: Counts per million
CPT: Carnitine palmitoyltransferase
Ct: Threshold cycle
CWDE: Cell wall degrading enzyme
DYN: Dynamin
E2F: Winged helix domain
ECHD: Enoyl-CoA hydratase
EFH: Other EF-hand protein
ELO: Translation elongation factor
ENOL: Enolase (phosphopyruvate hydratase)
FAT: Sodium-translocating F-ATPase
FBP: Fructose-1,6-bisphosphatase
FCP: Aspartate-based phosphatase
FDR: False discovery rate
FPF: Flagella protofilament ribbon
FPK: 6-phosphofructokinase
FPKM: Fragments per kilobase per million mapped reads
GAPDH: Glyceraldehyde-3-phosphate dehydrogenase
GLK: Glucokinase
GO: Gene ontology
GPH: Glycoside-pentoside-hexuronide transporter
GPI: Glucose-6-phosphate isomerase
GPX: Glutathione peroxidase
GRX: Glutaredoxin
GSR: Glutathione reductase
GST: Glutathione S-transferase
HADH: Hydroxy acyl-CoA dehydrogenase
HEL: DNA helicase
HLH: Helix-loop helix
HOM: Homeodomain
HSF: Heat shock factor
HTH: Helix-turn-helix
INI: Translation initiation factors
IRK: Inward rectifier potassium channel
KCS: Protein kinase catalytic subunit with cNMP domain
KRS: Adenylate kinase regulatory subunit
KVG: Potassium voltage-gated channel
LIG: DNA ligase
MCM: Minichromosome maintenance protein
MED: Transcription mediator
MFS: Major facilitator superfamily with sugar domain
MSI: Mechanosensitive ion channel
MYB: Myeloblastosis transcription factor
NFY: Nuclear transcription factor Y
NIT: Nitrate transporter
ORC: Origin replication complex protein
OTH: Other protein kinase
PC2: Polycystin 2
PCA: Principal component analysis
PDE: Phosphodiesterase
PEPCK: Phosphoenolpyruvate carboxykinase
PFK2: 6-Phosphofructose-2-kinase
PGK: Phosphoglycerate kinase
PGM: Phosphoglycerate mutase
PHO: Phosphate transporter
PLC: Phospholipase C
POL: DNA polymerase
PPDK: Pyruvate phosphate dikinase
PPM: Metallo-dependent protein phosphatase
PPP: Phosphoprotein phosphatase
PRI: Primase
PRX: Peroxiredoxin
PTP: Protein tyrosine phosphatase
PYC: Pyruvate carboxylase
PYK: Pyruvate kinase
REL: Translation release factors
RP: Ribosomal proteins
RPO: RNA polymerase subunit
RT-qPCR: reverse transcription quantitative polymerase chain reaction
STE: Sterile 20 kinase
SUL: Sulfate transporter
SWT: SWEET transporter
TAZ: Transcription adaptor putative zinc finger
TKL: Tyrosine kinase-like protein kinase
TMM: Trimmed mean of M-values
TOP: Topoisomerase
TPI: Triosephosphate isomerase
TRP: Transient receptor potential channel
TRR: Thioredoxin reductase
TRX: Thioredoxin
TUB: Tubby transcription factor
