# Supplementary material for: RNA-seq of life stages of the oomycete Phytophthora infestans reveals dynamic changes in metabolic, signal transduction, and pathogenesis genes and a major role for calcium signaling in development
Source: BMC Genomics. 2017 Feb 23;18:198. doi: 10.1186/s12864-017-3585-x (PMC5322657; doi:10.1186/s12864-017-3585-x)
Supplement: Additional file 1: — Libraries used for RNA-seq. (DOCX 64 kb) [file 12864_2017_3585_MOESM1_ESM.docx]

**Additional File 1.** Libraries for RNA-seq

| Isolate | Description | Replicates | Reads | Reads aligned | Per cent aligned |
| --- | --- | --- | --- | --- | --- |
| 1306 | nonsporulating mycelia | 2 | 44,997,268 | 39,754,274 | 88.3 |
|  | sporangia | 2 | 47,749,036 | 42,450,243 | 88.9 |
|  | chilled sporangia | 2 | 60,598,020 | 53,113,887 | 87.6 |
|  | zoospores | 2 | 48,851,738 | 43,168,609 | 88.4 |
|  | germinated cysts | 2 | 45,276,950 | 38,952,722 | 86.0 |
| 88069 | nonsporulating mycelia | 4 | 93,620,705 | 82,604,123 | 88.2 |
|  | sporangia | 2 | 49,014,975 | 43,694,459 | 89.1 |
|  | chilled sporangia | 4 | 107,542,905 | 95,510,266 | 88.8 |
|  | zoospores | 2 | 46,440,905 | 40,269,922 | 86.7 |
|  | chilled sporangia, 2-APB | 2 | 42,803,239 | 38,383,772 | 89.7 |
|  | chilled sporangia, trifluoroperazine | 2 | 47,188,764 | 36,170,497 | 76.7 |
|  | chilled sporangia, verapamil | 2 | 40,502,470 | 35,060,763 | 86.6 |
