# Supplementary material for: RNA-seq of life stages of the oomycete Phytophthora infestans reveals dynamic changes in metabolic, signal transduction, and pathogenesis genes and a major role for calcium signaling in development
Source: BMC Genomics. 2017 Feb 23;18:198. doi: 10.1186/s12864-017-3585-x (PMC5322657; doi:10.1186/s12864-017-3585-x)
Supplement: Additional file 4: — Phenotypic characterisation of PKS1 knockdowns. (DOCX 92 kb) [file 12864_2017_3585_MOESM4_ESM.docx]

**Additional File 5.** Phenotypes of *Pigbl1*-knockdown strains.

|  | Genetic background | | |
| --- | --- | --- | --- |
|  | Pigbl1  knockdowns | Control transformants | 1306  (wt) |
| Radial growth, rye media (mm/day) | 8.4±0.9 | 8.6±1.3 | 9.3±3.3 |
| Dry weight, rye media (mg at 10 d) | 45.5±0.7 | 45.8±1.0 | 47.5±0.7 |
| Radial growth, Plich media (mm/day)^g^ | 2.2±0.7 | 1.9±1.2 | 2.4±1.4 |
| Dry weight, Plich media (mg at 10 d)^h^ | 0.19±0.05 | 0.29±0.03 | 0.33 ±0.06 |
| Sporangium density (×10^4^ cm^-2^ at 10 d) | 32.3±8.8 | 35.0 ±3.4 | 45.5±14.9 |
| % sporangia releasing zoospores, 2 hr 10°C | 74.09±4.68 | 66.2 ±4.6 | 65.3±9.8 |
| % cysts forming appressoria, 16 hr 10°C | 54.9±3.4 | 56.9±2.1 | 56.3±2.9 |
| Sporangia per tomato leaflet (×10^5^ at 5 d) | 7.6±0.6 | 13.4±3.4 | 15.5±3.5 |
| Hyphal growth in tomato (arbitrary units) | 0.69±0.6 | 1.03±0.3 | 1.0±0.3 |

Data shown are averages ± standard deviations of the two knockdown strains, three control transformants (two expressing β-glucuronidase and one non-silenced transformant) and the wild type progenitor 1306, based on three replicates per strain. Growth rates (mm/day) were measured on agar media (10 days for rye media, 8 days for Plich), while dry weights were determined using broth media. Plant infections involving inoculating leaflets of tomato *cv.* Moneymaker at five sites with a 15 μl drop of 2 × 10^4^/ml sporangia. *In planta* growth was measured by qPCR with O8 primers, normalized to plant tissue weight and expressed in arbitrary units.
